# Socio-economic patterning of cardiometabolic risk factors in rural and peri-urban India: Andhra Pradesh children and parents study (APCAPS)

**DOI:** 10.1007/s10389-015-0662-y

**Published:** 2015-03-11

**Authors:** Vipin Gupta, Christopher Millett, Gagandeep Kaur Walia, Sanjay Kinra, Aastha Aggarwal, Poornima Prabhakaran, Santhi Bhogadi, Aniket Kumar, Ruby Gupta, D. Prabhakaran, K. Srinath Reddy, George Davey Smith, Yoav Ben-Shlomo, K. V. Radha Krishna, Shah Ebrahim

**Affiliations:** 1 Department of Anthropology, University of Delhi, New Delhi, 110007 India; 2 Faculty of Medicine, School of Public Health, Imperial College London, London, UK; 3 Public Health Foundation of India, Delhi NCR, India; 4 Department of Non-Communicable Disease Epidemiology, London School of Hygiene & Tropical Medicine, London, UK; 5 Centre for Chronic Disease Control, New Delhi, India; 6 MRC Integrative Epidemiology Unit, University of Bristol, Bristol, UK; 7 School of Social and Community Medicine, University of Bristol, Bristol, UK; 8 Indian Council for Medical Research, National Institute of Nutrition, Hyderabad, India

**Keywords:** Socioeconomic position, Status, Cardiovascular, Metabolic disease, Risk factors

## Abstract

**Aim:**

To assess the prevalence of cardiometabolic risk factors by socio-economic position (SEP) in rural and peri-urban Indian population.

**Subjects and methods:**

Cross-sectional survey of 3,948 adults (1,154 households) from Telangana (2010–2012) was conducted to collect questionnaire-based data, physical measurements and fasting blood samples. We compared the prevalence of risk factors and their clustering by SEP adjusting for age using the Mantel Hansel test.

**Results:**

Men and women with no education had higher prevalence of increased waist circumference (men: 8 vs. 6.4 %, *P* < 0.001; women: 20.9 vs. 12.0 %, *P* = 0.01), waist-hip ratio (men: 46.5 vs. 25.8 %, *P* = 0.003; women: 58.8 vs. 29.2 %, *P* = 0.04) and regular alcohol intake (61.7 vs. 32.5 %, *P* < 0.001; women: 25.7 vs. 3.8 %, *P* < 0.001) than educated participants. Unskilled participants had higher prevalence of regular alcohol intake (men: 57.7 vs. 38.7 %, *P* = 0.001; women: 28.3 vs. 7.3 %, *P* < 0.001). In contrast, participants with a higher standard of living index had higher prevalence of diabetes (top third vs. bottom third: men 5.2 vs. 3.5 %, *P* = 0.004; women 5.5 vs. 2.4 %, *P* = 0.003), hyperinsulinemia (men 29.5 vs. 16.3 %, *P* = 0.002; women 31.1 vs. 14.3 %, *P* < 0.001), obesity (men 23.3 vs. 10.6 %, *P* < 0.001; women 25.9 vs. 12.8 %, *P* < 0.001), and raised LDL (men 16.8 vs. 11.4 %, *P* = 0.001; women 21.3 vs. 14.0 %, *P* < 0.001).

**Conclusions:**

Cardiometabolic risk factors are common in rural India but do not show a consistent association with SEP except for higher prevalence of smoking and regular alcohol intake in lower SEP group. Strategies to address the growing burden of cardiometabolic diseases in urbanizing rural India should be assessed for their potential impact on social inequalities in health.

## Introduction

Non-communicable diseases (NCDs) are a growing burden on individuals and health systems globally (Di Cesare et al. 2013). While studies from high-income settings indicate that this burden disproportionately falls on individuals with lower socio-economic position (SEP), evidence from low and middle income (LMIC) settings is more mixed (Gupta et al. 2012; Zaman et al. 2012; Subramanian et al. 2013). For example, while cardiovascular (CVD) risk factors have been found to be more common in high SEP groups, CVD related mortality may be higher in low SEP groups (Subramanian et al. 2013). The scientific basis for the contrasting findings in LMICs is unclear, but may be partly due to differences in study design, use of self-reported versus objective measures of NCD risk (Vellakkal et al. 2013), data presentation and its interpretation (Subramanian et al. 2013).

In LMICs like India, it has been assumed that health transitions result from a rise in exposure to common risk factors for NCDs affecting mainly urban populations. However, epidemiological evidence shows a high mortality burden of NCDs (Joshi et al. 2006) and high levels of risk factors in rural India as well (Kinra et al. 2008), which is of concern, given that 68 % of Indian population live in rural areas where the reach of preventative health programmes can be low and where health care services remain substantially underdeveloped (Reddy et al. 2005; Vashishtha and Kumar 2013).

The social patterning of NCD risk factors also has important implications for individuals and households in LMICs, given the impoverishing impact of NCDs (Engelgau et al. 2012). Poor social protection, lack of universal health coverage and high out of pocket payments for health care will tend to exacerbate social inequalities due to NCDs. However, only a few studies have described the socio-economic patterning of NCD risk factors in rural India (Zaman et al. 2012), which is an important knowledge gap given an increasing focus on NCDs in the country and ongoing efforts to strengthen rural healthcare through the National Rural Health Mission (2011). Thus, the aim of this study is to examine the SEP patterning of cardiometabolic risk factors in a rural and peri-urban population.

## Materials and methods

### Study population

The index participant of Andhra Pradesh Children and Parents’ Study (APCAPS) includes children born during the time of the Hyderabad Nutrition Trial (1987–1990s), a cluster randomized trial, who were re-recruited in 2010–2012 along with their siblings and parents (Kinra et al. 2008, 2013). Since the index children have been followed by our team over time, there is strong rapport established in these villages, which resulted in >75 % response rate. The present analysis was undertaken on 3,948 adults (age ≥ 18 years) from 20 villages of the Rangareddy district of Andhra Pradesh. We excluded 51 participants with self-reported CHD and stroke to avoid bias. Ethical clearance for APCAPS was approved by the National Institute of Nutrition, Hyderabad and the Public Health Foundation of India, New Delhi.

### Study variables

#### Biochemical assays

Fasting glucose was measured on the same day of sampling using the glucose oxidase/peroxidase −4-aminophenazone-phenol enzymatic method (Trinder 1996) and two level controls from Randox Laboratory Ltd. (Crumlin City, UK). Other biochemical assays were performed in the Genetics and Biochemistry Laboratory (GBL) using Cobas311 autoanalyzer and reagents from Roche Diagnostics GmbH, Mannheim, Germany. Enzymatic calorimetric method was used to measure the total cholesterol (Roeschlau et al. 1974), triglycerides (Siedel et al. 1993) and serum high-density lipoprotein cholesterol (HDL-C; Matsuzaki et al. 1996). Low density lipoprotein cholesterol (LDL-C) level was estimated using standard the Friedewald-Fredrickson formula (Friedewald et al. 1972). Fasting insulin was assayed in serum samples on an e-411 autoanalyzer using an electrochemiluminescence immuno assay. The quality control for all biochemical assays were assessed by running two levels of internal controls (two pairs of duplicates and one sample from the previous batch) with every batch of 80 samples. The intra assay and inter assay coefficient of variation for all the parameters were <3 and <5 % respectively for all the assays. GBL participates in Randox International Quality Assessment Service regarding clinical chemistry parameters and United Kingdom National External Quality Control Assessment Service regarding insulin assay.

#### Anthropometry and physiological measurements

Height, weight, circumferences (waist and hip) and blood pressure were measured using standard instruments (Kinra et al. 2013). Anthropometric measurements were taken twice and the mean of the two measures was taken for all traits. The acceptable differences between the readings were ≤0.5 cm for height, 0.5 kg for weight and 0.5–1 cm for circumferences. After 5 min of relaxation, three consecutive readings on the right hand were taken both for systolic (SBP) and diastolic (DBP) blood pressure with a gap of 1 min in between, and then the average of the last two readings was considered for the analysis (or two acceptable readings). The acceptable difference between the two readings was ≤5 mmHg for diastolic and ≤7 mmHg for systolic blood pressure measurement.

#### Lifestyle factors

Information on tobacco smoking and alcohol use was gathered as part of a questionnaire. Details on tobacco smoking behavior, age at onset, duration of use and frequency were collected. Similarly, frequency and total consumption of present alcohol intake in the form of local spirits, branded spirits, wine and beer was recorded.

#### Socio-economic variables

Questionnaires were administered to gather information on age, sex, educational attainment, current occupation, current household circumstances and assets owned by the household from each participant. On the basis of education categories, participants who were illiterate or had no formal education were classified as having ‘No Education’ while participants with any level of formal education were classified as ‘Educated’. For occupation, participants were grouped as ‘Unskilled’ for unskilled manual workers, and ‘Skilled’ which included semi-skilled manual, skilled manual, skilled non-manual, semi-professional and professional. An ‘Other’ category was used to classify housewives, disabled, retired, students and unemployed individuals (1,033 individuals) and was not used for analyses based on occupation. The estimation of economic status of the household was based on the Standard of Living Index (SLI) that has been validated in Indian populations (Subramanian et al. 2006; Ebrahim et al. 2010). The information on house construction material, source of lighting, type of fuel, source of drinking water, type of toilet facility, separate kitchen, owning of agricultural land and some assets (clock, radio, TV, cycle, motorcycle, car, fridge, phone, water pump, cart, thresher and tractor) was used for the assessment of SLI. The weights developed by International Institute of Population Sciences in India for National Family Health Survey-3 (2007) were assigned to each variable accounting for maximum score of 42. This score was categorized into low (asset score ≤ 14), middle (asset score = 15–24) and high SLI (asset score >24).

#### Cardiometabolic risk factors

Diabetes was defined as either fasting plasma glucose (FPG) ≥126 mg/dl (WHO 2006) or self reported (doctor diagnosed) diabetes. Impaired fasting glucose was defined as FPG ≥ 100 mg/dl in males (American Diabetes Association guidelines 2012). Hyperinsulinemia was defined as fasting insulin >60 pmol (Borai et al. 2007). Hypertension was defined as either mean SBP ≥ 140 mmHg and mean DBP ≥ 90 mmHg , according to Joint National Committee on Prevention, Detection, Evaluation and Treatment of High BP guidelines (Chobanian et al. 2003), or self reported (doctor diagnosed) hypertension. Obesity was defined as BMI ≥ 25 kg/m^2^, overweight as BMI = 23.0–24.9 kg/m^2^ and underweight as BMI < 18 kg/m^2^ (Misra et al. 2009). High waist circumference (hWC) was defined as waist girth > 90 cm in males and >80 cm in females (Misra et al. 2009), while high waist-hip ratio (hWHR) was defined as WHR > 0.88 in males and >0.80 in females (Snehalatha et al. 2003; Misra et al. 2009). Hypercholesterolemia was defined as total cholesterol > 5.2 mmol/L, hypertriglyceridemia as triglycerides > 1.7 mmol/L, high LDL-C as LDL-C > 3.5 mmol/L and low HDL-C as HDL-C < 1 mmol/L in males and <1.3 mmol/L in females (Expert Panel on Detection, Evaluation, and Treatment of High Blood Cholesterol in Adults 2001). Current smokers included participants who smoked tobacco within the last 6 months. Regular alcohol intake included drinking any kind of alcohol (local spirits/branded/wine/beer) daily or on most days of the week.

### Statistical analysis

We classified participants into socioeconomic groups: education (1,932 no education/2,016 educated), occupation (1,877 unskilled/1,038 skilled) and SLI (780 low/2,375 middle/793 high). The prevalence of each of the 14 potential risk factor was tabulated for each of the socioeconomic groups and sex-wise comparisons between these groupings were made using the Mantel Haenszel test adjusting for age. The SEP patterning of co-occurrence of cardiometabolic risk factors was examined by creating a simple additive CVD risk score, summing smoking, hypertension, diabetes, hypercholesterolemia and increased waist-hip ratio. Additionally, we compared whether the age adjusted prevalence of having ≥3 risk factors differed by socio-economic status group in men and women using the Mantel Hansel test.

## Results

Our sample consisted of 3,948 adult participants (2,088 men and 1,860 women) residing in 20 villages. The mean age of men was 35.0 years and women was 35.5 years; 36.1 % of men and 63.3 % of women had no education; 41.9 % of men and 53.8 % of women worked in unskilled occupations; and 17.5 % of men and 22.2 % of women had low SLI. The association of the number of cardiometabolic risk factors, stratified by education, occupation and SLI, was higher in men than in women (Table 1).

**Table 1:** Prevalence of risk factors of cardiometabolic diseases in adults stratified by education

	Men (*N* 2,088)			Women (*N* 1,860)		
Risk factors/conditions	No education *N* (%)	Education *N* (%)	*P*	No education *N* (%)	Education *N* (%)	*P*
	754 (36.1)	1,334 (63.9)		1,178 (63.3)	682 (36.7)	
Impaired fasting glucose	141 (18.7)	182 (13.6)	0.284	181 (15.4)	47 (6.9)	*0.011*
Diabetes	41 (5.4)	40 (3.0)	*0.006*	63 (5.3)	14 (2.0)	0.718
Hyperinsulinemia	89 (11.8)	392 (29.4)	<*0.001*	221 (18.8)	185 (27.1)	0.204
Obesity	98 (13.0)	244 (18.3)	<*0.001*	241 (20.5)	102 (15.0)	*0.005*
Overweight	72 (9.5)	189 (14.2)	*0.009*	176 (14.9)	62 (9.1)	0.562
hWC	60 (8.0)	85 (6.4)	<*0.001*	246 (20.9)	82 (12.0)	*0.010*
hWHR	351 (46.5)	344 (25.8)	*0.003*	693 (58.8)	199 (29.2)	*0.042*
Hypertension	123 (16.3)	79 (5.9)	0.084	111 (9.4)	18 (2.6)	0.558
Low HDL-C	228 (30.2)	546 (40.9)	*0.001*	737 (62.6)	416 (61.0)	0.718
Hypertriglyceridemia	244 (32.4)	372 (27.9)	*0.009*	244 (20.7)	82 (12.0)	0.805
Hypercholesterolemia	175 (23.2)	246 (18.4)	*0.007*	285 (24.2)	89 (13.0)	0.404
High LDL-C	114 (15.1)	178 (13.3)	*0.049*	235 (19.9)	97 (14.2)	0.080
Current smoking	386 (51.2)	204 (15.3)	*<0.001*	3 (0.2)	1 (0.1)	0.250
Regular alcohol	465 (61.7)	433 (32.5)	*<0.001*	303 (25.7)	26 (3.8)	*<0.001*
≥3 risk factors	238 (31.5)	232 (17.4)	*0.001*	225 (19.1 %)	38 (5.6 %)	0.44

*hWC* high waist circumference, *hWHR* high waist-hip ratio, *HDL* high density lipoprotein, *LDL* low density lipoprotein, *P* value adjusted for age

Among men with no education, the prevalence of eight cardiometabolic risk factors (no education vs. education)—diabetes (5.4 vs. 3 %, *P* = 0.006), increased waist circumference (8 vs. 6.4 %, *P* < 0.001), waist-hip ratio (46.5 vs. 25.8 %, *P* = 0.003), hypertriglyceridemia (32.4 vs. 27.9 %, *P* = 0.009), hypercholesterolemia (23.2 vs. 18.4 %, *P* = 0.007), high LDL-C (15.1 vs. 13.3 %, *P* = 0.049), smoking (51.2 vs. 15.3 %, *P* < 0.001) and regular alcohol intake (61.7 vs. 32.5 %, *P* < 0.001)—was higher than in men with education (Table 1). Women with no education had higher prevalence of impaired fasting glucose (15.4 vs. 6.9 %, *P* = 0.01), obesity (20.5 vs. 15.0 %, *P* = 0.005), hWC (20.9 vs. 12.0 %, *P* = 0.01), hWHR (58.8 vs. 29.2 %, *P* = 0.04) and regular alcohol intake (25.7 vs. 3.8 %, *P* < 0.001) than those with an education.

Men working in unskilled occupations had high prevalence of hWHR (41.1 vs. 36.0 %, *P* < 0.001), hypertension (12.5 vs. 9.5 %, *P* < 0.001), smoking (42.4 vs. 22.2 %, *P* < 0.02) and regular alcohol (57.7 vs. 38.7 %, *P* = 0.001) intake than those working in skilled occupations but they had a lower prevalence of hyperinsulinemia, obesity, overweight, hWC, low HDL-C, hypertriglyceridDemia, hypercholesterolemia and high LDL-C (Table 2). Women working in unskilled occupation had high prevalence of hypercholesterolemia (20.7 vs .20.5 %, *P* = 0.02) and regular alcohol intake (28.3 vs. 7.3 %, *P* < 0.001).

**Table 2:** Prevalence of risk factors of cardiometabolic diseases in adults stratified by occupation

	Men (*N* 2,088)			Women (*N* 1,860)		
Risk factors/conditions	Unskilled *N* (%)	Skilled *N* (%)	*P*	Unskilled *N* (%)	Skilled *N* (%)	*P*
	875 (41.9)	833 (39.9)		1,002 (53.9)	205 (11.0)	
Impaired fasting glucose	148 (16.9)	127 (15.2)	0.751	131 (13.1)	29 (14.1)	0.085
Diabetes	44 (5.0)	29 (3.5)	0.189	35 (3.5)	7 (3.4)	0.332
Hyperinsulinemia	115 (13.1)	271 (32.5)	<*0.001*	170 (17.0)	54 (26.3)	0.079
Obesity	116 (13.3)	194 (23.3)	<*0.001*	170 (17.0)	37 (18.0)	0.066
Overweight	94 (10.7)	130 (15.6)	*0.040*	136 (13.6)	27 (13.2)	0.382
hWC	60 (6.7)	75 (9.0)	<*0.001*	165 (16.5)	32 (15.6)	0.093
hWHR	360 (41.1)	300 (36.0)	<*0.001*	539 (53.8)	88 (42.9)	0.628
Hypertension	109 (12.5)	79 (9.5)	<*0.001*	80 (8.0)	6 (2.9)	0.369
Low HDL-C	282 (32.2)	352 (42.3)	*0.009*	620 (61.9)	124 (60.5)	0.630
Hypertriglyceridemia	265 (30.3)	291 (34.9)	<*0.001*	199 (19.9)	21 (10.2)	0.097
Hypercholesterolemia	187 (21.4)	198 (23.8)	<*0.001*	207 (20.7)	42 (20.5)	*0.020*
High LDL-C	122 (13.9)	139 (16.7)	*0.003*	174 (17.4)	35 (17.1)	0.103
Current smoking	371 (42.4)	185 (22.2)	*0.020*	3 (0.3)	-	0.563
Regular alcohol	505 (57.7)	322 (38.7)	*0.001*	284 (28.3)	15 (7.3)	<*0.001*
≥3 risk factors	232 (26.5)	213 (25.6)	<*0.001*	155 (15.5)	24 (1.7)	0.240

*hWC* high waist circumference, *hWHR* high waist-hip ratio, *HDL* high density lipoprotein, *LDL *low density lipoprotein, *P* value adjusted for age

There was a higher prevalence of impaired fasting glucose (19.6 vs. 15.1 vs. 13.1 %, *P* = 0.05), current smoking (40.0 vs. 28.7 vs. 17.6 %, *P* < 0.001) and regular alcohol intake (48.5 vs. 44.8 vs. 33.8 %, *P* = 0.04) in men with low SLI compared with those with a middle and high SLI (Table 3). Further, lower prevalences of diabetes, hyperinsulinemia, obesity, overweight, hWHR, hWC, hypertriglyceridemia, hypercholesterolemia and high LDL-C were observed in men with low SLI than middle and low SLI (Table 3). Increased prevalence of regular alcohol intake was observed in women (26.4 vs. 17.0 vs. 9.1 %, *P* < 0.001) with lowest asset tertile, whereas high prevalence of diabetes, hyperinsulinemia, obesity, overweight, hWC, hWHR and high LDL-C were found among women with middle or high SLI (Table 3).

**Table 3:** Prevalence of risk factors of cardiometabolic diseases in adults stratified by standard of living index

	Men (*N* 2,088)				Women (*N* 1,860)			
	Standard of living index				Standard of living index			
Risk factors/conditions	Low *N* (%)	Middle *N* (%)	High *N* (%)	*P*	Low N (%)	Middle *N* (%)	High *N* (%)	*P*
*N* (%)	367 (17.6)	1,256 (60.1)	465 (22.3)		413 (22.2)	1,119 (60.2)	328 (17.6)	
Impaired fasting glucose	72 (19.6)	190 (15.1)	61 (13.1)	0.055	54 (13.1)	143 (12.8)	31 (9.4)	0.572
Diabetes	13 (3.5)	44 (3.5)	24 (5.2)	*0.004*	10 (2.4)	49 (4.4)	18 (5.5)	*0.003*
Hyperinsulinemia	60 (16.3)	284 (22.6)	137 (29.5)	*0.002*	59 (14.3)	245 (21.9)	102 (31.1)	<*0.001*
Obesity	39 (10.6)	194 (15.4)	110 (23.7)	<*0.001*	53 (12.8)	205 (18.3)	85 (25.9)	<*0.001*
Overweight	26 (7.1)	159 (12.7)	76 (16.3)	<*0.001*	40 (9.7)	156 (13.9)	42 (12.8)	*0.042*
hWC	10 (2.7)	94 (7.5)	41 (8.8)	<*0.001*	49 (11.9)	203 (18.1)	76 (23.2)	<*0.001*
hWHR	121 (33.0)	422 (33.6)	152 (32.7)	<*0.001*	174 (42.1)	568 (50.8)	150 (45.7)	<*0.001*
Hypertension	39 (10.6)	129 (10.3)	34 (7.3)	0.289	28 (6.8)	82 (7.3)	19 (5.8)	0.378
Low HDL-C	122 (33.2)	464 (36.9)	188 (40.4)	0.110	242 (58.6)	711 (63.5)	200 (61.0)	0.338
Hypertriglyceridemia	99 (27.0)	355 (28.3)	162 (34.8)	<*0.001*	69 (16.7)	205 (18.3)	52 (15.8)	0.374
Hypercholesterolemia	63 (17.2)	251 (20.0)	107 (23.0)	<*0.001*	84 (20.3)	225 (20.1)	65 (19.8)	0.208
High LDL-C	43 (11.4)	172 (13.7)	78 (16.8)	*0.001*	58 (14.0)	204 (18.2)	70 (21.3)	<*0.001*
Current smoking	147 (40.0)	361 (28.7)	82 (17.6)	<*0.001*	1 (0.2)	3 (0.3)	-	0.688
Regular alcohol	178 (48.5)	563 (44.8)	157 (33.8)	*0.046*	109 (26.4)	190 (17.0)	30 (9.1)	<*0.001*
≥3 risk factors	86 (23.4)	276 (21.9)	108 (23.2)	*0.001*	45 (10.9)	175 (15.6)	43 (13.1)	*0.005*

*hWC* high waist circumference, *hWHR* high waist-hip ratio, *HDL* high density lipoprotein, *LDL* low density lipoprotein, *P* value adjusted for age

**Fig. 1:**
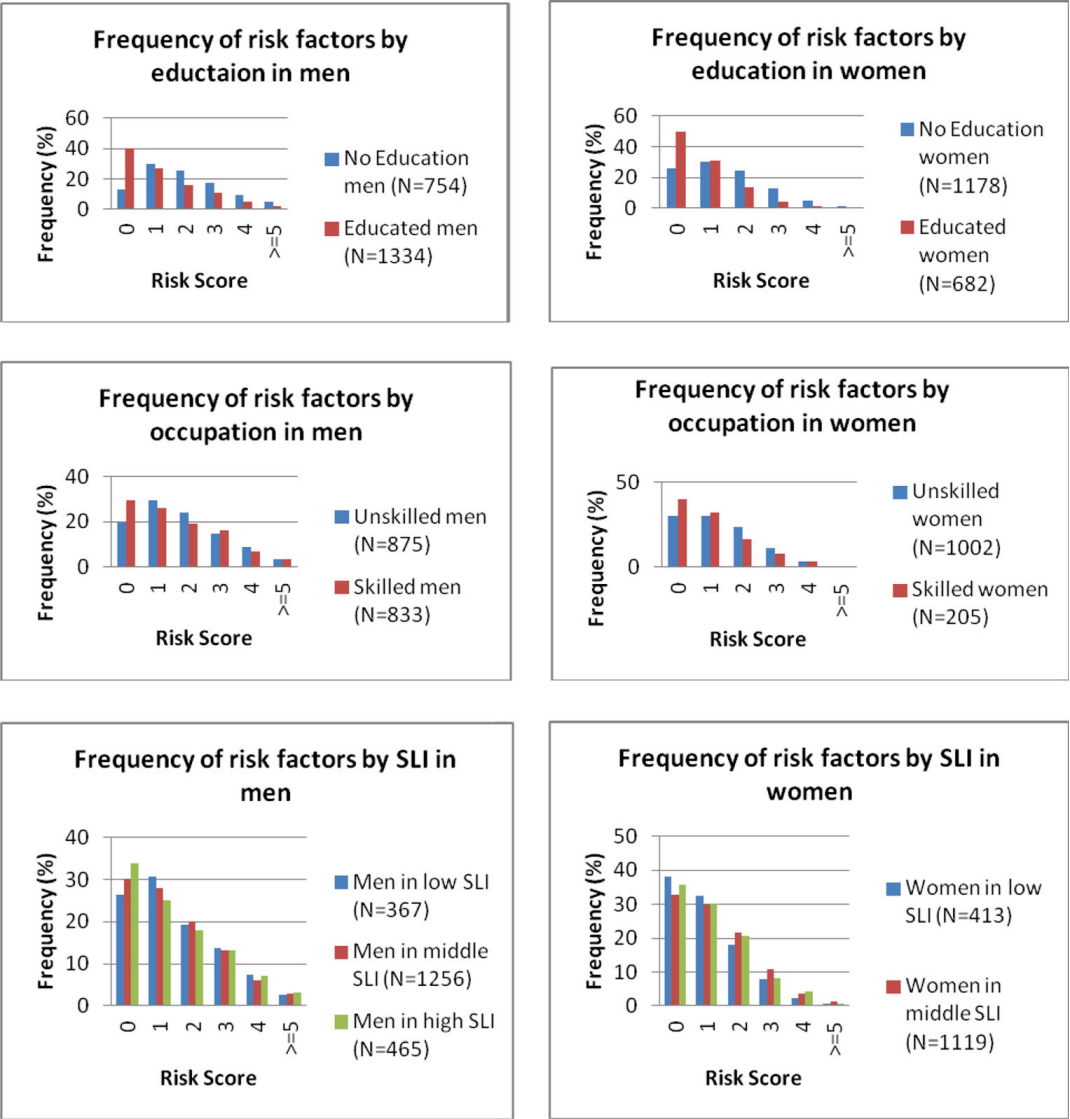
Graph showing frequency of count of risk factors related to cardio-metabolic diseases stratified by indicators of socioeconomic position in APCAPS. Risk factors (8): impaired fasting glucose; diabetes, overweight/obesity, hypertriglyceridemia, High LDL, current smoking. high WHR, hypertension

The percentages of men and women with zero to five or more CVD risk factors are presented in Fig. 1. The age-adjusted prevalence of ≥3 risk factors was higher in men with no education (31.5 vs. 17.4 %, *P* = 0.001), unskilled men (26.5 vs. 25.6 %, *P* < 0.001) and low SLI category (23.4 vs. 21.9 vs. 23.2 %, *P* = 0.001) compared to men with education, skilled occupation and middle or high SLI, respectively. Women with middle SLI had higher prevalence of ≥3 risk factors (10.9 vs. 15.6 vs. 13.1 %, *P* = 0.005) in comparison to low or high SLI. There were no significant differences in the age-adjusted prevalence of ≥3 risk factors in women by education and occupation.

## Discussion

This study identified a high prevalence of cardiometabolic risk factors in rural and peri-urban India. The social patterning of individual risk factors varied with associations differing between men and women with the measure of SEP used. Our finding that smoking is more common in low SEP groups is consistent with previous studies conducted in rural India (Zaman et al. 2012; Kinra et al. 2008), in other LMICs (Hosseinpoor et al. 2011) and high income countries (Hiscock et al. 2012). In contrast, hyperinsulinemia, obesity and overweight were more common among higher SEP groups, corroborating previous published evidence on CVD in India (Samuel et al. 2012; Subramanian et al. 2013).

While the co-occurrence of cardio-metabolic risk factors, as well as its SEP distribution, has been studied in urban Indians (Ramachandran et al. 1998; Gupta et al. 2012), it has not been previously explored in a rural and peri-urban populations. We found that men and women with no education were more likely to have multiple risk factors compared with those with some education (Fig. 1), a similar pattern to that previously observed in an urban population (Gupta et al. 2012). This might be due to undergoing transition towards urbanization of the studied villages that may influence multiple risk factors. Further, clustering of 3 or more risk factors was associated in men with no education, unskilled occupations and low SLI after adjusting for age (Tables 1, 2 and 3) which is contrast to Europeans where co-occurrence of risk factors did not explain social inequalities in cardiovascular disease (Ebrahim et al. 2004).

### Strengths and limitations of the study

APCAPS is a population-based cross-sectional study conducted in rural Indian settings which includes participants from 20 villages from the state of Andhra Pradesh. The present study does not have the limitations of previous attempts on SEP-based distribution of various risk factors from rural India like relatively small sample (i.e., 1221 rural participants, Samuel et al. 2012), or sampling limited to educational status (Gupta et al. 1994) or use of income instead of more reliable asset score (Zaman et al. 2012). The high response rates per village (>75 %) was another strength of the present study. Our study examined 14 risk factors related to cardiometabolic diseases of which 12 were biologically measured with high level of quality control. Further, the accuracy of direct measurement of income is difficult to obtain in LMICs (Howe et al. 2008); therefore, we used asset score (Ferguson et al. 2003) as a reliable alternative to income and consumption expenditure (Filmer and Pritchett 2001; Howe et al. 2008). We used a simple additive score to assess co-occurrence of risk factors. We did not assess the predicted long-term CVD risk in this study population using risk scores such as Framingham, which have been used in Indian populations (Jeemon et al. 2011) but have not been validated in prospective Indian cohorts. Moreover, WHO CVD risk factor scoring system has been recommended for use in LMICs and, while published charts are available, the equations have never been published. As use of fixed dose combination therapies is now recommended for prevention of CVD, developing appropriate tools for Indian populations should have a high priority.

### Importance of study findings

In light of limited studies on cardiometabolic risk factors in rural India, the current study has added information about their prevalence, co-occurrence and social patterning in this setting. Our findings indicate an inconsistent relationship between socio-economic position and specific markers of cardiometabolic risk which needs consideration when planning NCD prevention and management strategies in these settings. However, our findings on co-occurrence of CVD risk factors in lower SEP groups, largely driven by overweight, high blood pressure and smoking, indicate that future CVD burdens may disproportionately affect lower SEP groups. Current WHO policy is to target absolute cardiovascular risk rather than focusing on specific risk factors; however, validation of the predictive value of risk scoring and evaluation of implementing such strategies is needed. Ongoing monitoring of the socio-economic patterning of cardiometabolic risk will be important to assess the impact of existing social and health policies in the country. These include poverty alleviation projects such as the *National Rural Employment Guarantee Programme*, which aims to increase employment opportunities in rural areas, and the *National Rural Health Mission*, which aims to strengthen the provision of health care in rural settings.

### Conclusions

Cardiometabolic risk factors are common in rural dwelling Indian adults. There is considerable variation in the relationship between SEP and cardiometabolic risk in this setting, with smoking and alcohol use more common in low SEP groups and diabetes, hyperinsulinemia, overweight and high LDL-C more common among higher SEP groups. Strategies to address the growing burden of cardiometabolic disease in rural India should be assessed for their potential impact on social inequalities in health.
